# Physicochemical Properties, Antioxidant Capacity and Bioavailability of Whey Protein Concentrate-Based Coenzyme Q10 Nanoparticles

**DOI:** 10.3390/antiox13121535

**Published:** 2024-12-15

**Authors:** Yuxue Sun, Jiafei Liu, Xiaowen Pi, Alyssa H. Kemp, Mingruo Guo

**Affiliations:** 1 College of Food Science, Northeast Agricultural University, Harbin 150030, China; yuxue20@neau.edu.cn (Y.S.); 13361617637@163.com (X.P.); 2 Department of Nutrition and Food Sciences, College of Agriculture and Life Sciences, University of Vermont, Burlington, VT 05405, USA; ahkemp@uvm.edu

**Keywords:** coenzyme CoQ10, whey protein, physicochemical properties, antioxidant capacity, bioavailability

## Abstract

Coenzyme Q10 (CoQ10) is a powerful antioxidant. However, the poor water solubility and low bioavailability still remain challenges for its application. An embedded delivery system of CoQ10 based on whey protein concentrate (WPC) and polymerized whey protein concentrate (PWPC) was prepared, and the physicochemical properties, antioxidant capacity and bioavailability were characterized in this study. Both groups of nanoparticles showed a particle size distribution from 241 to 331 nm in the protein-to-CoQ10 mass ratio range of 100:1 to 20:1. In addition, the minimum polydispersity index value was observed at the mass ratio of 20:1. Differential scanning calorimetry and Fourier transform infrared spectra analysis revealed that the CoQ10 was successfully dispersed in the WPC and PWPC particles through hydrophobic interaction in both groups in addition to the hydrogen bond present in the WPC group. All nanoparticles exhibited irregular spherical or aggregate structure in the transmission electron microscopy diagram. The PWPC-based nanoparticles showed a slightly higher antioxidant capacity than that of the WPC, and both values were significantly higher than that of its corresponding physical mixture and free CoQ10 (*p* < 0.05). The results of the simulated gastrointestinal digestion experiments denoted that these two nanoparticles could protect CoQ10 from gastric digestion and then deliver it to the intestine. Compared with its free state, the bioavailability of CoQ10 embedded in WPC and PWPC increased by nearly 7.58 times and 7.48 times, respectively. The data indicated that WPC and PWPC could be effective delivery carriers to enhance the bioavailability of active substances like CoQ10.

## 1. Introduction

Coenzyme Q10 (CoQ10), a fat-soluble antioxidant synthesized in vivo, is one of the substances involved in electron transport chain and aerobic respiration in eukaryotic mitochondria. It can activate the nutrition of human cells and cell energy, thereby improving human immunity, enhancing anti-oxidation. Positive effects were also observed in the CoQ10 interventions for cardiovascular disease, Alzheimer’s disease, chronic kidney disease, depression, and diabetes [1,2,3,4,5,6]. Unfortunately, CoQ10 synthesized in vivo is known to decrease substantially with age [7]. In addition, multiple factors, including insufficient intake, reduced synthesis (genetic defects, cardiovascular disease, diabetes, cancer, etc.) and excessive consumption (excessive fatigue, metabolic excitement, acute shock), may lead to CoQ10 deficiency, under which, exogenous CoQ10 could be an alternative to maintain the normal level in the body. CoQ10 has low toxicity and will not cause serious adverse reactions to the human body. A reported intake of up to 1200 mg/day is the observed safe level for CoQ10 [8]. Pharmacokinetic studies indicated that exogenous CoQ10 did not affect the biosynthesis of endogenous CoQ10, and no accumulation was observed in plasma or tissues after withdrawal [9]. The regulatory risk assessment did not lead to any hazards associated with supplemental CoQ10 [10]. Therefore, CoQ10 is widely used in dietary supplements and food additives. Notably, CoQ10 is poorly absorbed in the gastrointestinal tract [11], and the resulting low bioavailability is still a challenge for CoQ10 application.

There have been many efforts to improve the bioavailability of CoQ10 through delivery systems based on improving the dispersibility/water solubility, protecting CoQ10 from environmental effects and promoting absorption and penetration, including liposomes [12,13], nanoemulsions [14,15], nanoparticles [16,17], micelles [18,19], solid dispersion [20,21,22,23] and a self-microemulsifying delivery system [24]. The application of these delivery systems has brought some improvements to the bioavailability of CoQ10, but there are still shortcomings, such as the complex preparation process and high cost. A pharmacokinetics study of 54 healthy volunteers taking commercially available supplements of CoQ10 showed that the delivery system affected its relative bioavailability. In terms of the overall bioavailability, the solubilizing agent was superior to the oil-containing dispersion and crystalline CoQ10 [25]. Utilizing natural food materials as wall materials to encapsulate CoQ10 may be an a convenient, safe and effective way of delivery to improve its hydrophilicity and bioavailability after oral administration. Chen et al. [26] suggested that food proteins, especially milk proteins, are effective carriers for enhancing the water dispersion, stability and even biological accessibility of CoQ10. Whey protein, which is stable in the presence of pepsin but degrades rapidly under trypsin, is a promising choice [27]. The water solubility of CoQ10 encapsulated in two milk-derived whey proteins (β-lactoglobulin and lactoferrin) was increased by 60 times and 300 times, respectively, compared with CoQ10 alone [28]. In another study, CoQ10 was loaded into high internal phase Pickering emulsions based on whey protein gel particles, and the results showed a significantly improved bioaccessibility [29]. Better water solubility and bioaccessibility were also observed in a previous study on whey protein isolation-based CoQ10 nanoparticles [30]. Although there have been some studies on the complexes of CoQ10 and whey proteins, information on whey protein concentrate (WPC)-based CoQ10 is not yet comprehensive. WPC, which has a lower cost and wider range of applications in industrialization than other high-purity whey protein products (such as whey protein isolation), requires more attention. Polymerized WPC (PWPC) is formed by the polymerization of WPC. During this process, WPC molecules are linked to each other through covalent bonds (such as disulfide bonds) or non-covalent bonds (such as hydrogen bonds, hydrophobic interactions, etc.) to form a larger polymer structure, showing a network-like or cluster-like structural morphology, which gives it different properties from WPC. PWPC is also a potential choice as a wall material for embedding active substances.

In this study, CoQ10 was encapsulated in WPC and PWPC at different mass ratios to prepare nanoparticles. The physicochemical properties, in vitro antioxidant properties, stability and bioavailability of the nanoparticles during gastrointestinal digestion were investigated. These data could provide evidence for the application of WPC in CoQ10.

## 2. Materials and Methods

### 2.1. Materials

WPC (80% purity) was obtained from Fonterra Co., Ltd. (Auckland, New Zealand). CoQ10 (>99% purity) was obtained from Xiamen Kingdomway Co., Ltd. (Xiamen, China). Pepsin (3000 units/mg), pancreatin (200 units/mg) and bile salts were purchased from Sigma-Aldrich (St. Louis, MO, USA). Other chemicals were all analytical grade, and all solutions were obtained using Milli-Q treated water.

### 2.2. Preparation of Nanoparticles

The nanoparticles (NPs) of WPC-CoQ10 and PWPC-CoQ10 were prepared according to the methods reported previously with some modifications [16,30]. WPC powder was dissolved in water to a final concentration of 100 mg/mL, followed by fully hydrating overnight at 4 °C. The resulting solution was further performed by adjusting the pH to 7.0, heating at 85 °C for 30 min, and finally cooling to room temperature in an ice-water bath to obtain the PWPC solution. CoQ10 was dissolved in ethyl acetate to a concentration of 10 mg/mL. The CoQ10 solution (5, 6.25, 8.33, 12.5 and 25 mL) was mixed with 50 mL of the WPC/PWPC solution to attain final mass ratios of protein to CoQ10 of 100:1, 80:1, 60:1, 40:1 and 20:1, respectively. The mixed solution was treated with a high-speed disperser at 12,000 rpm for 2 min. Then, high pressure homogenization was performed twice at 60 MPa. Finally, ethyl acetate was removed by a vacuum rotary evaporator (0.001 MPa, 45 °C), and deionized water was added to keep the sample volume constant (50 mL). The resulting NPs were directly used or freeze-dried for further analysis. The physical mixture of WPC/PWPC with CoQ10 (mass ratio of protein to CoQ10 at 20:1) for antioxidant analysis, and in vitro gastrointestinal digestion was prepared by adding 250 mg of CoQ10 to 50 mL of the WPC/PWPC solution and then treated through a high-speed disperser at 12,000 rpm for 2 min. CoQ10, used as a control, was the ethyl acetate solution of CoQ10. Aluminum foil was used to protect the sample from CoQ10 decomposition during the whole process.

### 2.3. Characterization of the Physicochemical and Structural Properties

#### 2.3.1. Particle Size and Zeta Potential Measurement

The particle size, zeta potential and polydispersity index (PDI) of all samples were measured using a Zetasizer (Nano-ZS90, Malvern Instruments Ltd., Worcestershire, Malvern, UK). All samples were diluted with deionized water to 1 mg/mL (protein concentration). Parameters for the particle size measurement were set to a refractive index of 1.455, an absorbance of 0.001, and a sample shading rate of 10–15%. The zeta potential value was obtained under the following parameters: the equilibrium time was 120 s, each sample was measured three times, and each measurement was set to run automatically in the range of 10–100 times. The PDI value was procured according to the particle size.

#### 2.3.2. Encapsulation Efficiency (EE) and Loading Capacity (LC) Measurement

First, 0.5 mL of WPC/PWPC-CoQ10 NPs solution was mixed with 1.5 mL dichloromethane, followed by vortexing for 10 s. After that, an obvious stratification of the organic phase and the aqueous phase appeared, and the free CoQ10 existed in the organic phase. Subsequently, the organic phase was transferred to a new centrifuge tube directly or in combination with a dropper. This procedure was repeated 3 times. The collected organic phase was then removed using a nitrogen evaporator (N-EVAPTM 112, Organomation Associates Inc., Berlin, MA, USA), and the remaining CoQ10 was redissolved with 5 mL n-hexane. The absorbance at 275 nm was determined using an ultraviolet spectrophotometer. The content of CoQ10 was obtained based on the standard curve. The EE and LC values of CoQ10 are calculated as follows:

$$EE\left(\%\right)=(1-\frac{Free\;content\;of\;CoQ10}{Added\;content\;of\;CoQ10})\times 100$$

$$LC\left(\%\right)=\frac{Mass\;of\;encapsulated\;CoQ10}{Total\;mass\;of\;nanoparticles}\times 100$$

The total mass of the nanoparticles in the formula refers to the sum of the protein mass and the CoQ10 mass added in the preparation of the corresponding nanoparticles. These two parameters of the different samples were calculated by the volume of the NPs solution used for measurement (0.5 mL), as well as the protein concentration in the solution (100 mg/mL) and the mass ratios of protein to CoQ10 (100:1, 80:1, 60:1, 40:1 and 20:1), respectively.

#### 2.3.3. Differential Scanning Calorimetry (DSC) Measurement

The thermal properties of the WPC/PWPC-CoQ10 NPs were analyzed using a differential scanning calorimeter (DSC3, Mettler Toledo, Greifensee, Switzerland). Each sample (5 mg) was placed in an aluminum plate sealed with a perforated aluminum cover. The heat procedure was increased from 25 °C to 150 °C at a constant rate of 10 °C/min. The nitrogen purge rate was 50 mL/min.

#### 2.3.4. Fourier Transform Infrared Spectra (FTIR) Measurement

The interaction between CoQ10 and WPC/PWPC was studied by infrared spectroscopy. The measurement conditions were as follows: the number of scans was 64, the wave number ranged from 500 to 4000 cm^−1^, and the resolution was 4 cm^−1^.

#### 2.3.5. Microstructure Measurement

The microstructure of the WPC/PWPC-CoQ10 NPs was observed using transmission electron microscopy (TEM). Briefly, the samples were moderately diluted with deionized water and dried in the air after being adsorbed on the copper mesh. Then, the microstructure of the samples at different magnifications (3000, 6000 and 10,000) was observed at an accelerating voltage of 100 kV.

### 2.4. Characterization of In Vitro Antioxidant Activity 

WPC/PWPC-CoQ10 NPs, the physical mixture of WPC/PWPC with CoQ10, were prepared under the mass ratio of protein to CoQ10 of 20:1 (the concentration of CoQ10 was 5 mg/mL). The in vitro antioxidant activity of these NPs, the physical mixture and free CoQ10 (5 mg/mL, dissolved in ethyl acetate) was characterized and compared.

#### 2.4.1. Determination of the ABTS Free Radical Scavenging Activity

The working solution was obtained by mixing ABTS solution (7 mmol/L) and potassium persulfate solution (2.45 mmol/L) in the same volume, and it was stored overnight in the dark at 25 °C. The working solution was then diluted with PBS buffer (5 mmol/L, pH 7.4) to an absorbance of 0.70 ± 0.02 at 734 nm for further use. The samples of NPs, the physical mixture and the free CoQ10 solution were diluted with deionized water to CoQ10 concentrations of 12.5, 25, 50 and 100 μg/mL, and then, 2.5 mL was mixed with 2.5 mL of the diluted working solution for reaction at room temperature for 18 min. The absorbance at 734 nm was measured with an ultraviolet photometer. A blank control was performed using PBS buffer solution. The ABTS free radical scavenging activity of the sample was calculated by the following formula:

$$The\;ABTS\;free\;radical\;scavenging\;activity\left(\%\right)=(\frac{A_{control}-A_{sample}}{A_{control}})\times 100$$

#### 2.4.2. Determination of the Total Reducing Power

Samples of NPs, the physical mixture and free CoQ10 solution were diluted with deionized water to a CoQ10 concentration of 50, 100, 200 and 400 μg/mL, respectively. Then, 1.0 mL of the diluted sample was mixed with 2.5 mL of PBS (200 mmol/L, PH 6.6) and 2.5 mL of potassium ferricyanide (10 mg/mL) and heated in a water bath at 50 °C for 20 min. After that, 2.5 mL trichloroacetic acid (100 mg/mL) was added, and centrifugation at 1500× *g* was performed for 10 min. The 2.5 mL supernatant was mixed with 2.5 mL of deionized water and 0.5 mL of ferric chloride (1 mg/mL). The absorbance of the obtained solution at 700 nm was measured after being incubated in the dark for 10 min.

### 2.5. Characterization of the In Vitro Digestion Properties

#### 2.5.1. Simulation of Gastro-Intestinal Digestion

First, 5 mL of the sample was mixed with 45 mL of HCl (0.1 mol/L), followed by shaking in the incubator for 10 min (37 °C, 100 rpm/min). The gastro-intestinal digestion was carried out according to the following steps. Gastric digestion: The pH of the solution was adjusted to 2.0, and 10 mg pepsin was added to the simulated gastric juice (SGF) with continuous shaking at 250 rpm/min, with sampling of 10 mL at 0 min, 15 min, 30 min, 45 min and 60 min, respectively. The obtained samples were placed in an ice bath, then adjusted to pH 7 and frozen for subsequent analysis. Intestinal digestion: First, 250 mg of pig bile salt was added to the final gastric digestive product, and then, its pH was adjusted to 7.0. After 10 min of shaking, 20 mg of trypsin was added to simulate the digestion of intestinal fluid (SIF). The digested samples (10 mL) were taken at 60 min and 120 min of intestinal digestion, respectively. The enzymatic hydrolysis reaction was terminated by adjusting the pH value to less than 6 (acidic). The resulting samples were frozen at −20 °C for further analysis.

#### 2.5.2. SDS-Page Profile

The digested samples were diluted with deionized water to a protein concentration of 5 mg/mL. The resulting sample (40 μL) was mixed with 10 μL SDS-PAGE loading buffer and heated at 100 °C for 2 min. Then, 5 μL of the mixed sample was added to the gel (5 wt % concentrated gel and 12 wt % separation gel containing 0.1 wt % SDS) for strip separation. Image Lab^TM^ (Version 4.0) Software was used to analyze the SDS-PAGE gels after staining with Coomassie brilliant blue R250.

#### 2.5.3. Bioaccessibility Measurement of CoQ10

A total of 2 mL of the intestinal final digestion samples was centrifuged at 10,000× *g* for 30 min, and the mixed micelle phase (supernatant) was collected. After that, 2 mL of the final intestinal digestion samples and the mixed micelles were dissolved in ethanol and n-hexane solution (1:1, *v*/*v*), respectively, and then centrifuged at 2250× *g* for 5 min. CoQ10 was extracted by diluting the supernatant with n-hexane, and then, the absorbance of the CoQ10 solution at 275 nm was determined by ultraviolet spectrophotometer. The concentration of CoQ10 was calculated based on the standard curve of CoQ10, and the bioaccessibility was calculated according to the following formula.

$$Bioaccessibility\left(\%\right)=\frac{C_{M}}{C_{D}}\times 100$$

In the formula, *C_M_* and *C_D_* represent the CoQ10 concentration in mixed micelles and intestinal final digestion samples, respectively.

#### 2.5.4. Particle Size, Zeta-Potential and PDI Measurement

The particle size, zeta-potential and PDI of the samples obtained during gastrointestinal digestion were measured via the same method described in Section 2.3.1.

#### 2.5.5. Microstructure Characterization

The microstructure of the samples obtained during gastrointestinal digestion was measured via the same method described in Section 2.3.5.

### 2.6. Statistical Analysis

The experiment was carried out three times, and the results were expressed as the mean and standard deviation. One-way analysis of variance (ANOVA) and Duncan’s test were performed using SPSS software (version 21.0) to compare the data. The statistical significance threshold of *p* value was set to lower than 0.05.

## 3. Results and Discussion

### 3.1. Physicochemical and Structural Properties

#### 3.1.1. Particle Size, Zeta-Potential and PDI Values

**Figure 1:**
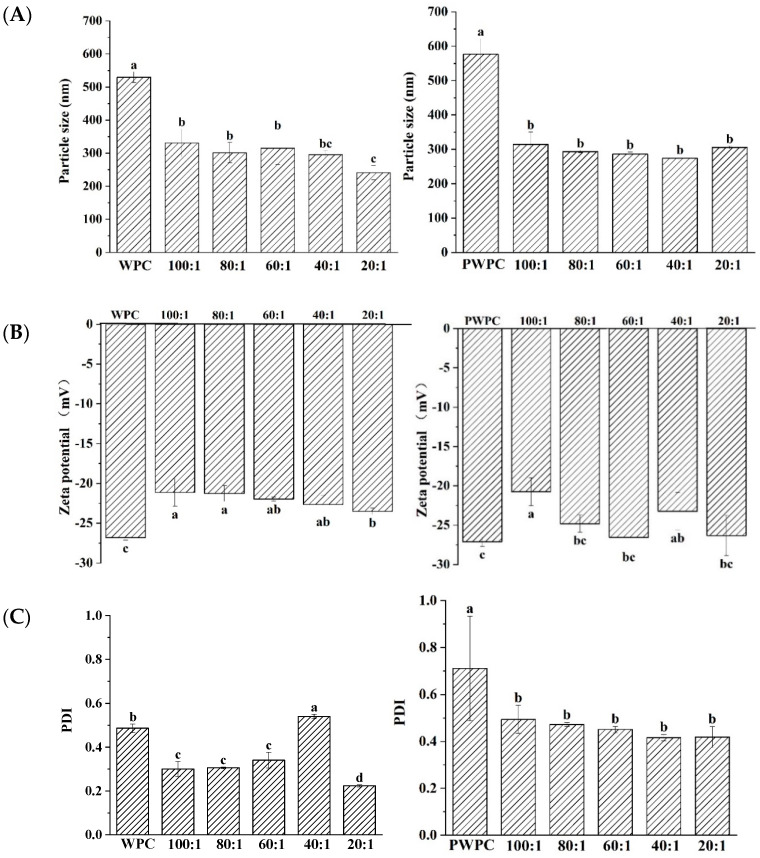
Particle size (**A**), Zeta potential (**B**) and polydispersity index (**C**) of WPC− and PWPC−based CoQ10 NPs with different mass ratios (protein to CoQ10 from 100:1 to 20:1). Statistical analysis of values between groups was denoted by lowercase letters above volumes, where different lowercase letters suggested a statistical difference at *p* < 0.05.

The particle size, zeta-potential and PDI values of WPC-CoQ10 and PWPC-CoQ10 NPs were characterized (Figure 1). The results showed that a uniform and stable nano-system was prepared in both the WPC and PWC. In the range of the protein-to-CoQ10 mass ratio of 20:1 to 100:1, the minimum particle size of WPC-CoQ10 NPs appeared at a 20:1 ratio of 241 nm. For the PWPC group, the smallest particles were observed at 40:1, but there was no significant difference from those of 20:1 (272 nm vs. 305 nm, *p* > 0.05) (Figure 1A). Similar results were found for the Zeta potentials and PDI value. As shown in Figure 1B, the absolute value of the Zeta potential of the two NPs was the highest at 20:1 across all the ratios (−23 mV and−26 mV, respectively). A high surface charge endowed high stability to these two nano-systems [31]. The minimum PDI values for the WPC-CoQ10 and PWPC-CoQ10 NPs occurred at 20:1 (0.2236 and 0.41873, respectively) (Figure 1C), illustrating the good homogeneity displayed in the nano-system [32].

#### 3.1.2. EE and LC

**Figure 2:**
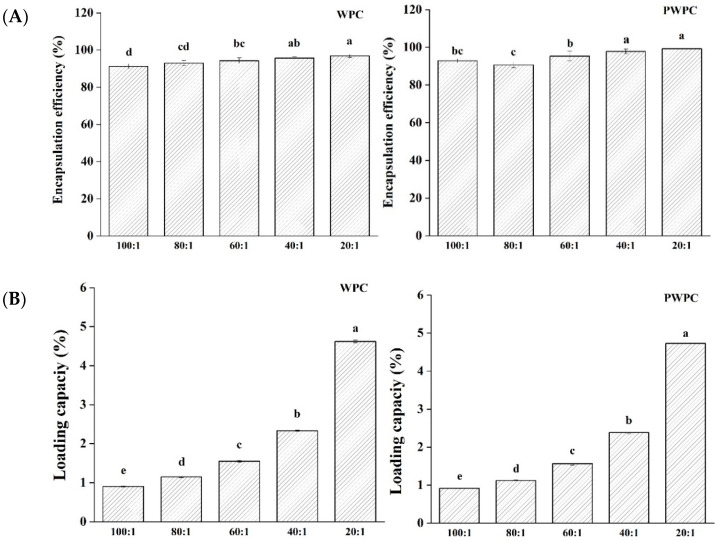
Encapsulation efficiency (**A**) and loading capacity (**B**) of WPC− and PWPC−based CoQ10 NPs with different mass ratios (protein to CoQ10 from 100:1 to 20:1). Statistical analysis of values between groups was denoted by lowercase letters above volumes, where different lowercase letters suggested a statistical difference at *p* < 0.05.

Figure 2 shows the EE and LC values of CoQ10 in the WPC and PWPC based nano-systems. As can be seen from Figure 2A, all the samples had high EE values (all over 90.7%) in the ratio range from 20:1 to 100:1, implying that only a small fraction (less than 9.3%) of CoQ10 was free rather than successfully embedded in the protein matrix. The highest EE value of the WPC group was 96.88%, and that of the PWPC group was 99.28%, both of which appeared at the ratio of 20:1. With the increase in CoQ10, the LC values of the two groups increased significantly (*p* < 0.05) (Figure 2B). As expected, the values peaked at the ratio of 20:1, reaching 4.620% (WPC group) and 4.729% (PWPC group), respectively, confirming the excellent loading capability of WPC and PWPC for CoQ10.

#### 3.1.3. DSC and Fourier Transform Infrared Spectroscopy (FTIR)

**Figure 3:**
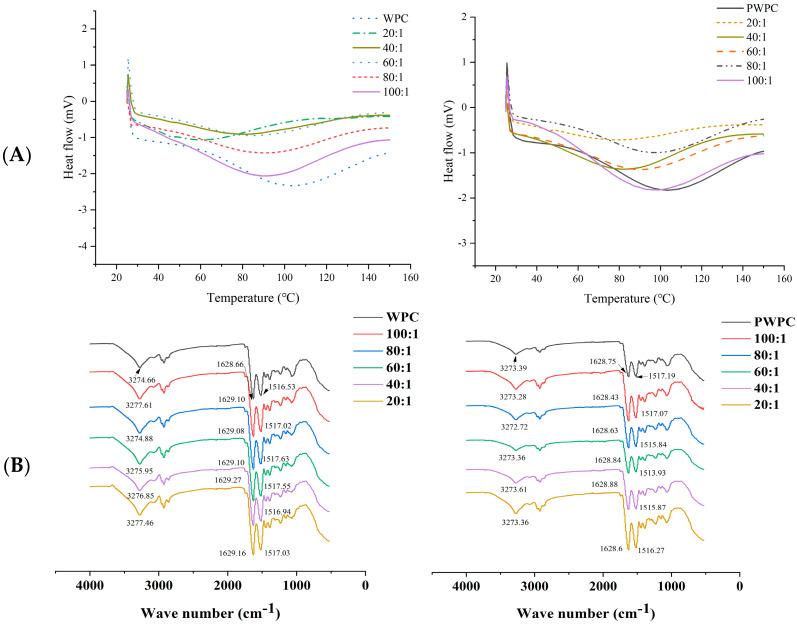
DSC (**A**) and FTIR spectra (**B**) of WPC− and PWPC−based CoQ10 NPs with different mass ratios (protein to CoQ10 from 100:1 to 20:1).

The DSC thermogram of the native WPC/PWPC demonstrated a wide endothermic peak between 50 and 150 °C with the peak temperature about 103.5 °C (Figure 3A). With the increase in the CoQ10 loading, the denaturation temperature range and peak temperature of the WPC-CoQ10 and PWPC-CoQ10 NPs decreased obviously, and no melting peak (~52.89 °C) of free CoQ10 occurred in either, implying that CoQ10 was dispersed in the WPC/PWPC based nano-system in an amorphous form [33,34]. This was agreement with the results observed in PWPI-CoQ10 NPs in a previous study [30]. The FTIR spectra analysis (Figure 3B) showed that the WPC displayed the peaks of -OH (3274.66 cm^−1^), amide I (C=O vibration, 1628.66 cm^−1^) and amide II (C-N vibration, 1516.53 cm^−1^), all of which blue-shifted after binding to CoQ10, suggesting the formation of intramolecular hydrogen bonds and hydrophobic interactions between WPC and CoQ10. While for PWPC-CoQ10 NPs, a red-shift in the amide II (C-N vibration) peak was observed compared with native PWPC, speculating that the PWPC was structurally changed, and hydrophobic interactions were mainly formed. CoQ10 could form complexes with different proteins through hydrophobic interactions [26]. Hydrogen bonds and hydrophobic interactions were also reported in other delivery systems of fat-soluble bioactive components including curcumin and piperine [35].

#### 3.1.4. Transmission Electron Microscopy (TEM)

**Figure 4:**
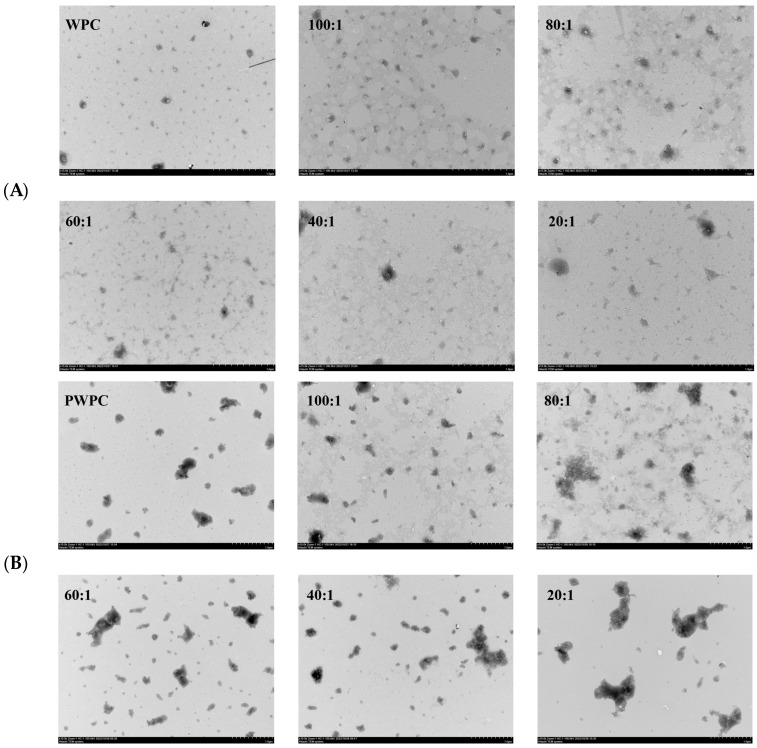
Microstructure of WPC−(**A**) and PWPC−based (**B**) CoQ10 NPs with different mass ratios (protein to CoQ10 from 100:1 to 20:1).

The microstructures of the WPC− and PWPC−based nano-systems were investigated, and their TEM images are shown in Figure 4. All the NPs with different mass ratios displayed an irregular spherical or aggregate structure. As shown in Figure 4A, the WPC particles exhibited a regular spherical structure that spans about 10–100 nm. With the loading of CoQ10, aggregates appeared, and the particle size increased with the increase in the CoQ10 loading. The majority of NPs were at a size of roughly 100 nm, and a few larger aggregates about 200 nm were observed when the mass ratio reached 20:1. For the PWPC group (Figure 4B), the aggregate structure (about 100–600 nm) was clearly observed in PWPC particles compared with the WPC, which was due to the aggregation caused by thermal modification. Similar results were reported in WPI under thermal modification [30]. A morphology of vermicular aggregates was then presented with a size of 100–300 nm after loading CoQ10. When the PWPC/CoQ10 ratio was 20:1, some larger aggregates of about 800 nm were observed. There was a difference between the particle size observed by TEM and that by the Zetasizer, which can be attributed to several reasons. One is the different principles of the two measuring methods [36]. The particle size measured using the Zetasizer is a three-dimensional massive dynamic particle distribution (volume-based), while TEM mainly observes the particle morphology, which is based on a small point-surface static particle measurement (area-based). Therefore, different particle sizes were observed between the two methods, especially for irregular aggregate particles. Another reason may that the state of the sample particles was different, in which the Zetasizer measured the hydration particles, whereas dehydrated particles were observed via TEM.

### 3.2. Antioxidant Activity

**Figure 5:**
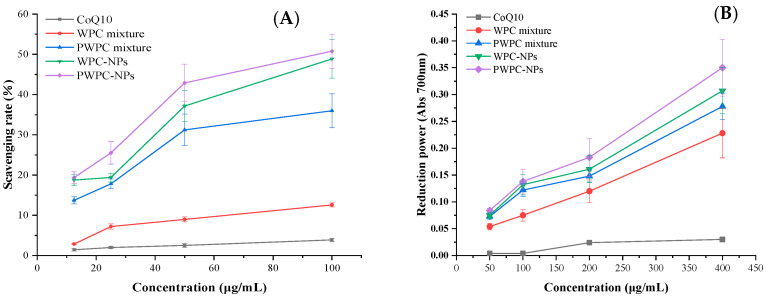
ABTS radical scavenging rate (**A**) and reducing power (**B**) of free CoQ10, physical mixtures and WPC- and PWPC-based CoQ10 NPs with a mass ratio of 20:1.

NPs and physical mixtures of CoQ10 based on WPC and PWPC were prepared, and their ABTS free radical scavenging activity and total reduction power values were compared with free CoQ10 (Figure 5). As expected, both the radical scavenging activity and the total reducing force increased with the increase in the CoQ10 loading, the two indexes of both NPs were higher than their corresponding physical mixtures, respectively, and the free CoQ10 was the lowest. As shown in Figure 5A, the ABTS free radical scavenging activities of PWPC−NPs, WPC−NPs, PWPC mixtures, WPC mixtures and free CoQ10 were 19.38 ± 1.49%, 18.74 ± 1.38%, 13.73 ± 0.89%, 2.87 ± 0.13% and 1.44 ± 0.34%, respectively, at a CoQ10 concentration of 12.5 μg /mL. It then rose to 50.76 ± 4.28%, 48.86 ± 4.78%, 35.98 ± 4.21%, 12.58 ± 0.48% and 3.89 ± 0.41%, respectively, when the CoQ10 concentration rose to 100 μg/mL. The results of the total reduction power values were consistent with changes in the ABTS free radical scavenging activity. The strongest total reduction force was found in PWPC-NPs, which showed the highest absorbance of 0.350 ± 0.053, followed by WPC-NPs (0.307 ± 0.043). The PWPC mixture and WPC mixture ranked third and fourth with 0.278 ± 0.024 and 0.228 ± 0.046, respectively. The absorbance of free CoQ10 was only 0.030 ± 0.005 (Figure 5B). All the above results indicated that the antioxidant activity of CoQ10 was significantly enhanced by the prepared NPs, which may be related to the improvement in the water solubility of CoQ10. Similar effects were reported in nano-systems of CoQ10 prepared from whey protein isolation [30], polyethylene glycol monostearate micelles [37] and dextran [38].

### 3.3. In Vitro Digestibility

#### 3.3.1. Stability of Nanoparticles During Digestion

**Figure 6:**
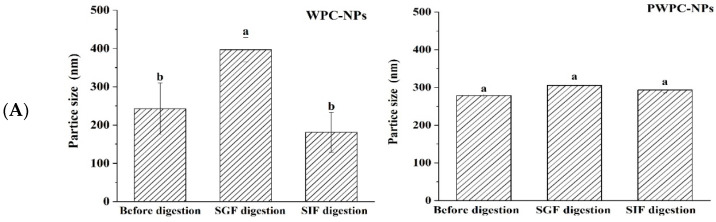

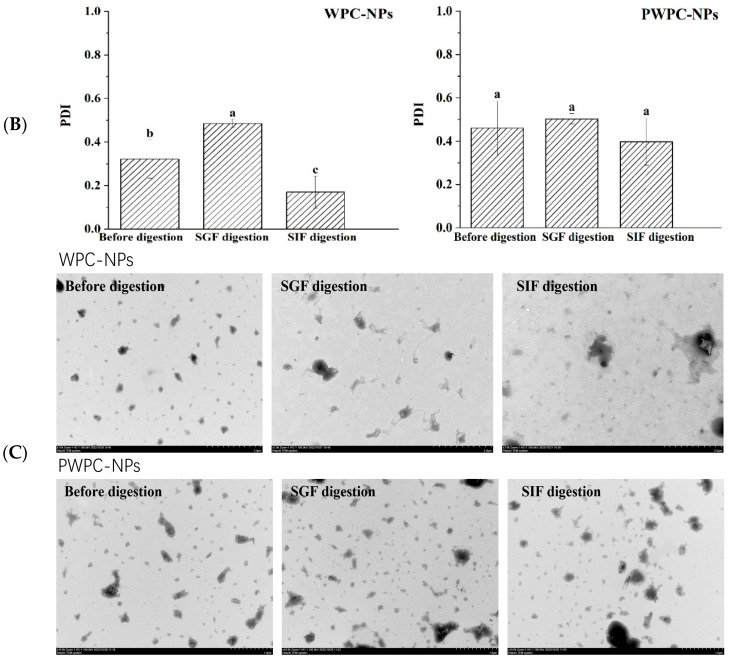

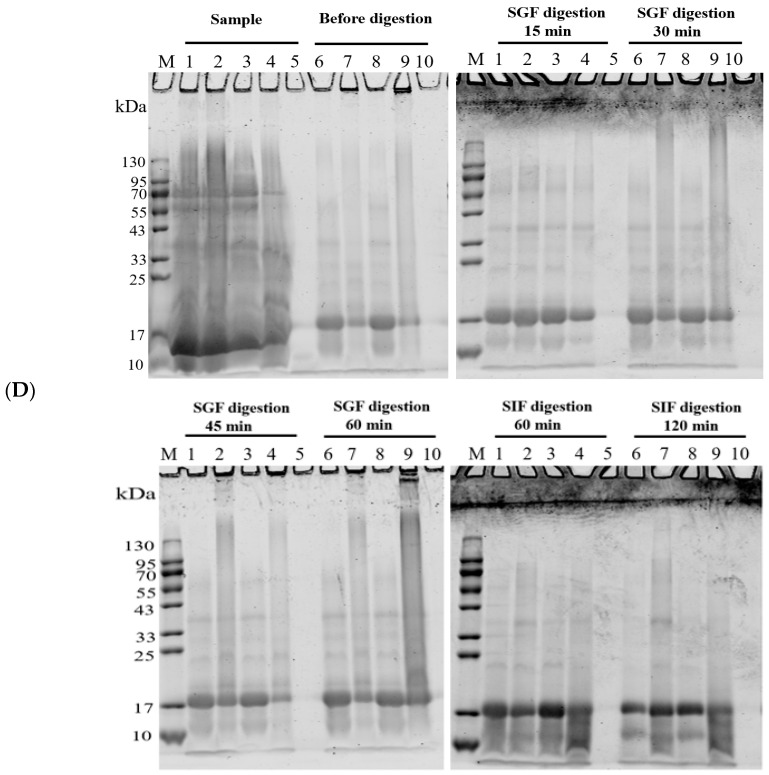
Particle size (**A**), polydispersity index (**B**), microstructure (**C**) and SDS-PAGE patterns (**D**) of WPC- and PWPC-based CoQ10 NPs during gastrointestinal digestion. For the SDS-PAGE patterns (**D**), Lands 1 and 6 = physical mixture of WPC-CoQ10, lands 2 and 7 = physical mixture of PWPC-CoQ10, lands 3 and 8 = WPC-CoQ10 NPs, lands 4 and 9 = PWPC-CoQ10 NPs and lands 5 and 10 = free CoQ10 (in ethyl acetate). “Sample” refers to the original sample without the addition of SGF. Statistical analysis of values between groups was denoted by lowercase letters above volumes, where different lowercase letters suggested a statistical difference at *p* < 0.05.

Figure 6 shows the performance of the WPC-and PWPC-based CoQ10 NPs with the mass ratio of protein to CoQ10 at 20:1 (the CoQ10 concentration was 5 mg/mL) during gastrointestinal digestion. As shown in Figure 6A, the particle size of the WPC-CoQ10 NPs first increased from 242.6 ± 28.71 to 397.20 ± 18.28 nm during gastric digestion, then decreased to 181.03 ± 25.76 nm in subsequent intestinal digestion. For the PWPC-CoQ10 NPs, similar trends, but not significantly (*p* > 0.05), seemed to be observed throughout the whole digestion, which can be explained by the stability improvement in the whey protein after thermal polymerization [39]. The PDI value increased during gastric digestion, indicating a wider molecular-weight distribution of the particles, and the distribution was then uniform after entering the intestine with a decreased value (Figure 6B). Both NPs aggregated in the acidic environment of the stomach and then trypsinized into small particles in the intestine, accompanied by a portion of the digested protein cross-linked and recombined by bile salts (Figure 6C). The SDS-PAGE protein profiles revealed that changes in the protein bands occurred mainly in SIF digestion, with varying degrees of downshift in the whey protein bands at 60 min and 120 min of SIF digestion (Figure 6D). These results indicated that both WPC- and PWPC-based NPs could resist gastric digestion and deliver CoQ10 into the intestine successfully, which was attributed to the antipepsin advantage of β-lactoglobulin in the whey protein [40]. Previous studies suggested that substances present in bile can interact with CoQ10 molecules to form micelles, which transport CoQ10 to the surface of intestinal absorption cells and then release CoQ10 for absorption [41,42].

#### 3.3.2. Bioaccessibility of CoQ10

**Figure 7:**
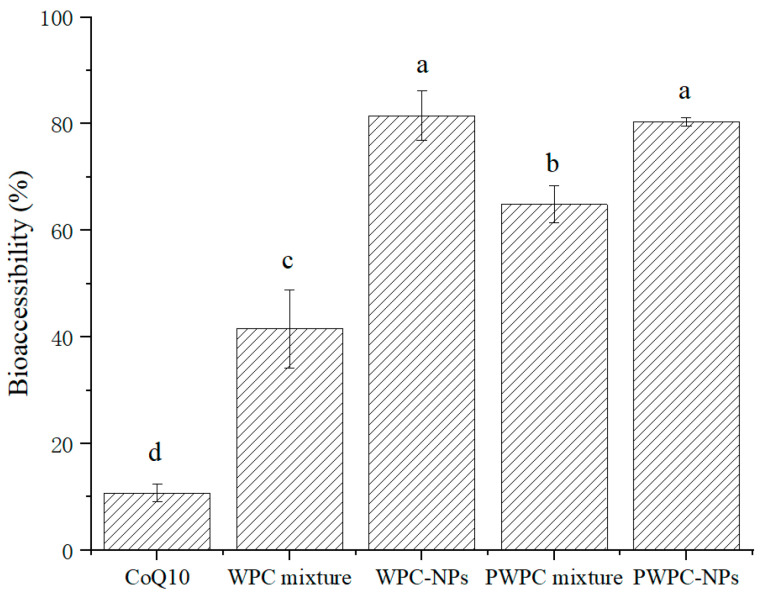
Bioaccessibility of free CoQ10, physical mixtures of CoQ10 with WPC and PWPC, WPC-, and PWPC-based CoQ10 NPs after gastrointestinal digestion. Statistical analysis of values between groups was denoted by lowercase letters above volumes, where different lowercase letters suggested a statistical difference at *p* < 0.05.

Figure 7 shows the bioaccessibility of WPC-and PWPC-based CoQ10 NPs and their corresponding physical mixtures. The highest bioaccessibility was observed in WPC- and PWPC-based NPs, followed by the two physically mixed samples. As expected, the lowest bioaccessibility occurred in the free CoQ10, which is consistent with the results reported previously [26]. Compared with free CoQ10 (10.75 ± 1.70%), the bioaccessibility of the WPC-CoQ10 and PWPC-CoQ10 NPs increased to 81.53 ± 4.62% and 80.37 ± 0.84%, respectively, about 7.58 times and 7.48 times that of free CoQ10, indicating that the embedding of CoQ10 by WPC and PWPC could successfully achieve the release of CoQ10 in the intestinal tract. WPC-CoQ10 NPs showed a slightly higher bioaccessibility than the PWPC group, which may be induced by their smaller particle size [43]. Compared to the large particles, larger specific surface areas were exhibited in the smaller particles, which allowed CoQ10 to be more readily dissolved and released from the particle surface, thereby improving its bioavailability. A similar phenomenon was observed in a previous study on the construction of lutein ternary NPs using goat milk casein [44]. The relative bioavailability of CoQ10 loaded on lecithin was increased by 176.6% [15]. In another study, a 1.7-fold higher enhancement was reported in the CoQ10 liquid nano-emulsion than the crystalline CoQ10 [45]. In recent years, the bioavailability of CoQ10 formulations has also been studied in vivo [46,47,48]. The improved bioavailability of WPC-CoQ10 NPs and PWPC-CoQ10 NPs in the present study was also validated by in vivo rat-based experiments, in which the CoQ10 concentrations in plasma were significantly higher in rats given a suspension of WPC/PWPC-CoQ10 NPs than in free CoQ10 (*p* < 0.05), and no significant difference was observed between the WPC group and the PWPC group (*p* > 0.05) [27]. The higher bioavailability presented in our study indicates a better delivery performance of WPC/PWPC for CoQ10.

## 4. Conclusions

WPC-CoQ10 and PWPC-CoQ10 NPs were successfully prepared in this study. The entrapment efficiency of all the NPs was above 90.7%. After CoQ10 was encapsulated, its antioxidant activity was significantly improved (*p* < 0.05). It seemed that both CoQ10 NPs could reach the lower part of GI tract successfully under simulated gastrointestinal digestion. Compared with free CoQ10, the bioavailability of WPC- and PWPC-encapsulated CoQ10 increased 7.58- and 7.48-fold, respectively. The results indicated that both WPC and PWPC are excellent wall materials for embedding CoQ10 to improve its bioavailability.

## Data Availability

The original contributions presented in the study are included in the article; further inquiries can be directed to the corresponding authors.
